# Tobacco extract is a chemotaxis repellent for *C. elegans*

**DOI:** 10.17912/micropub.biology.002188

**Published:** 2026-06-22

**Authors:** Baraa J. Abdelghne, Zeinab K. Zreik, Ashley N. Carter, Gwendolyne K. Aguilar, Griselda Morales, Dave Ramirez, Nicole Bradon, Chloe L. Golde, Lauren A. O'Connell

**Affiliations:** 1 BIO161 Organismal Biology Lab, Stanford University, Stanford, California, United States; 2 Department of Biology, Stanford University, Stanford, California, United States

## Abstract

Plants synthesize compounds that interact with animal nervous systems to avoid predation, but the behavioral effects and underlying mechanisms of commonly encountered plant natural products remain understudied. We tested whether cannabis, ginger root, pepper tree, saffron, and tobacco extracts elicit chemotaxis responses in the nematode *Caenorhabditis elegans* . We found that tobacco extract repels *C. elegans* , whereas the other tested plant extracts did not have a significant effect on behavior. These experiments were conducted in an undergraduate laboratory course, providing both research training and new insights into plant–animal interactions.

**Figure 1. Tobacco ( Nicotiana tabacum ) extract is a chemotaxis repellent for Caenorhabditis elegans:** **(A)** Crushed plant samples were extracted with methanol (tobacco photo was taken separately and published in Wu et al., 2026). **(B)** Chemotaxis plates (35 mm) were divided into nonadjacent experimental (E) quadrants and solvent (X) quadrants; worms were placed in the center gray circle (7.9 mm). **(C)** The chemotaxis response of *C. elegans* for each plant extract is shown as boxplots, with boxes representing the first and third quartiles, the median as a solid black line, and the dots as individual chemotaxis assays. There was a significant difference in chemotaxis response among plant extracts (GLMM: χ²(5) = 22.163, p < 0.001). Only tobacco extract was significantly different from the solvent (t = -2.683, p _adj_ = 0.045).

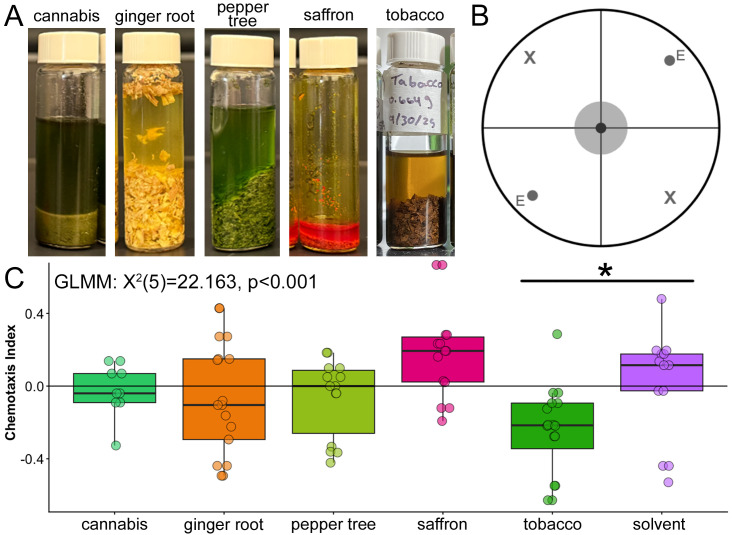



## Description

Behaviors between organisms can be influenced by bioactive small molecules (Howe and Jander, 2008). Among the many molecular cues that exist in nature, some plants produce chemicals capable of influencing neuronal activity in humans and other animals (Alrashedy and Molina, 2016). The nematode *Caenorhabditis elegans* is an important model system for understanding chemosensation via olfactory pathways, given its simple nervous system, genetic toolkit, and well-characterized behavioral responses to specific chemical cues (Bargmann, 2006). Yet, how *C. elegans* responds to medicinal plant compounds is understudied (Fryer et al., 2024), which could help inform how medicinal plants influence target neural pathways.

We asked how *C. elegans* responds to five commonly used psychoactive and medicinal plants containing bioactive molecules that influence nervous system function in diverse animals. Tobacco ( *Nicotiana tabacum* ) produces nicotine, an alkaloid that binds to nicotinic acetylcholine receptors in many animals (Benowitz, 1996). Cannabis ( *Cannabis sativa* ) produces cannabinoids that interact with neuromodulatory receptors and ion channels (Oakes et al., 2017). Ginger root ( *Zingiber officinale* ) contains gingerols and shogaols that activate sensory neurons (Zhang et al., 2021). Saffron ( *Crocus sativus* ) contains crocins and safranal, which have neuromodulatory and antioxidant properties (Schmidt et al., 2007). Finally, the pepper tree ( *Schinus molle* ) produces essential oils rich in terpenoids with known antimicrobial and irritant effects (Rebolledo et al., 2021). We hypothesized that, given their use in traditional or herbal medicine, these plant extracts would elicit a chemotaxis response in *C. elegans* .

Plants were obtained from local vendors or gardens, and their natural products were extracted with methanol ( Figure 1A ), which extracts a wide range of polar to moderately polar compounds, including alkaloids, some terpenoids, and phenolic compounds like tannins (Altemimi et al., 2017). Then, plant extracts were used as olfactory stimuli in *C. elegans* chemotaxis assays ( Figure 1B ), where nematodes can display repulsion, attraction, or indifference. We predicted that *C. elegans* would respond with attraction or repulsion to plants with natural products that were moderately polar, like nicotine in tobacco, gingerols in ginger, and crocins and safranal in saffron. On the other hand, we predicted that plant extracts with more nonpolar natural products, like cannabinoids in cannabis, would not induce a chemotaxis response.

Wild-type worms ( CGC1 ) responded differently to various plant extracts. Specifically, tobacco repelled the worms compared to the solvent, whereas the other plants did not significantly differ from the solvent control group. This response is consistent with the presence of alkaloids like nicotine, which can activate nicotinic acetylcholine receptors and disrupt neuromuscular signaling (Benowitz, 1996; Rand, 2007). Prior research in *C. elegans* has shown that nicotine exposure can influence behavior through cholinergic and TRP-channel pathways, with effects that depend on dose and context (Feng et al., 2006). As nicotine can impair locomotion and reduce survival, the avoidance response observed here is consistent with avoiding compounds that interfere with motor control (Rand, 2007). Of note, most studies use pure nicotine, whereas we used extract from tobacco leaves, which likely contains many natural products, and we cannot determine whether nicotine and/or other compounds are responsible for the repellent effect observed here. Future studies with single compounds would be necessary to fully understand how the leaf extract components regulate behavior.

Other plant extracts exhibited more variable or neutral responses that were not significantly different than solvent control and did not align with our predictions based on the extraction method and polarity of natural products. Ginger root and pepper tree extracts have bioactive terpenes and phenolic compounds that can influence sensory pathways in other organisms (Garzoli et al., 2019). For example, pepper tree volatiles have a repellent effect in flies and moths (Patocka and Almeida, 2017), although we did not observe a response in *C. elegans* . In contrast, saffron showed a non-significant positive trend, suggesting mild attraction, potentially due to volatile aromatic compounds such as safranal (Schmidt et al., 2007). In line with our predictions, cannabis extract produced a near-neutral response, which may be due to inefficient extraction of cannabinoids in methanol or the underlying molecular mechanisms of action, as cannabinoids primarily modulate internal neuromodulatory pathways rather than directly driving chemotaxis behavior (Oakes et al., 2017; Oakes et al., 2019). Additionally, there are key differences in endocannabinoid receptors between *C. elegans* and vertebrate animals, where these molecules may be transduced differently in the nervous system (Estrada-Valencia et al., 2023). Together, these results suggest that *C. elegans* integrates multiple chemical cues within complex extracts, leading to variable behavioral outputs depending on compound composition.

In summary, our results demonstrate that tobacco extract is a natural chemotaxis repellent for *C. elegans* . Future experiments could analyze the chemical profile of the tobacco extract and test individual molecules in chemotaxis assays at varying concentrations to determine which set of molecules is responsible for the repellent effect. We cannot rule out that the other plants tested here can impact *C. elegans* chemotaxis behavior, given different chemical extraction and processing steps for the plants or concentrations of the stimuli (Yoshida et al., 2012), and further research is warranted. As plant alkaloids and terpenes influence sensory perception and behavior through conserved receptors (Changeux, 2010), this study further supports the use of *C. elegans* as a model system for uncovering biologically active plant compounds.

## Methods

*Worm strains*

The wild-type *Caenorhabditis elegans* strain used in this study was sourced from the Caenorhabditis Genetics Center (CGC, reference strain formally known as PD1074 ) at the University of Minnesota. Worms were maintained in 20°C incubators and synchronized by bleaching adults to isolate eggs. Eggs were then grown on nematode growth media plates spread with OP50 *Escherichia coli* , following standard protocols (Stiernagle, 2006). Roughly 500 embryos were placed on each growth plate, and one growth plate was generated for every two chemotaxis assay plates. Hatched eggs were maintained at 20°C for four days until the population reached a young adult stage. Worms were then utilized for chemotaxis assays.

*Plant extracts and compounds*

Students obtained ginger root ( *Zingiber officinale* ), saffron ( *Crocus sativus* ), cannabis ( *Cannabis sativa* ), and tobacco ( *Nicotiana tabacum* ) from local stores, and collected pepper tree ( *Schinus molle* ) leaves on the Stanford University campus. The plant species was identified using the Seek nature identification app by iNaturalist (California Academy of Sciences, version 2.17.4; November 10, 2025). Plant leaves were washed with deionized water, patted dry with a paper towel, and left to dry overnight at 60°C (Margie et al., 2013). The plant material was then weighed, cut into small pieces, and ground into a fine powder using a mortar and pestle. The powder was transferred into pre-weighed glass vials, and the plant mass was calculated by subtracting the pre-weighed vial mass from the filled vial mass. Vials were filled to no more than half their volume, and methanol was added to approximately twice the volume of the plant material. Methanol was filtered to remove plant debris, transferred to a clean glass vial, and then evaporated using nitrogen gas. Remaining plant extracts were resuspended in dimethyl sulfoxide (DMSO) to a 10 mg/mL concentration for use in chemotaxis assays.

*Chemotaxis assays*

Undergraduate students conducted chemotaxis assays in a laboratory classroom as previously described (Alfonso et al., 2023; Lopez et al., 2024; Gaerlan et al., 2025) following standard procedures (Bradon et al., 2024). Chemotaxis assays were conducted across two laboratory sessions (10 assays per plant per class session). Students were unaware of the chemical stimulus being tested until class data was submitted to the instructor. Chemotaxis plates [5mM KPO4 (pH 6), 1mM CaCl2, 1mM MgSO4, 2% agar on a 35 mm plate] were divided into four quadrants by taping a transparent template to the bottom of each plate ( Figure 1B ). Then, 5 μL plant extract (10 mg/mL) was placed on dots located in two non-adjacent quadrants (E, experimental), while 5 μL of DMSO (solvent) was placed on X marks in the other two quadrants. Control plates had DMSO (solvent) placed in all four quadrants. Plates were incubated for 30 min while worms were prepared for assays.

Worms were removed from the growth plates (one growth plate per two assays) using chemotaxis assay buffer [5mM KPO4 (pH 6), 1mM CaCl2, 1mM MgSO4] and moved to a microfuge tube. The worms were washed two to three times with chemotaxis buffer. During these washes, worms were allowed to sink to the bottom of the tube for 2-3 min before the supernatant was removed and more chemotaxis buffer was added. After the final wash, approximately 100 μL of buffer was left covering the worms. Then, 2 μL of 0.5 M sodium azide solution was applied to each of the assay's quadrant spots to eventually paralyze the worms at their choice locations. Immediately after, 20 µL worms (with the goal of 75-100 worms per plate) were pipetted into the center of each chemotaxis plate (gray 7.9mm circle, Figure 1B ), where they were allowed to roam for 30 min undisturbed. Excess liquid was removed with KimTech Wipes if worms were not moving away from the center after five minutes of incubation. Worms were manually counted under a dissecting microscope using a tally counter. Worms in the center area of overlapping quadrants were not counted to avoid including dead worms in the dataset. Across all plates, the average number of worms per plate was 78 (+/- 32 standard deviation).

*Data analysis*

Plates that noted experimental errors, such as tilting or worms clumping in the center or rim, were not included in the analysis. Plates with fewer than 25 or more than 110 worms were also excluded from analysis, as low counts reduce the reliability of chemotaxis estimates and high counts can reflect overcrowding or counting error. For each assay, we calculated the chemotaxis index [CI = (number of worms in experimental quadrants - number of worms in solvent quadrants) / total worms scored], where roughly zero is neutral, positive values indicate attraction, and negative numbers indicate repulsion. Data analysis and visualization were performed in RStudio (version 2025.09.1+401) running R (version 4.3.0). We used generalized linear mixed models to test for differences in chemotaxis behavior across plant extracts using the glmmTMB package (version 1.1.7, Brooks et al., 2017). The proportion of worms in the experimental quadrants (number of worms in experimental quadrants / total worms scored) was used as the dependent variable, with compound as a fixed effect and session as a random effect to account for variation across assay runs. Model diagnostics were evaluated using the DHARMa package (version 0.4.6, Hartig, 2024) to test for dispersion and confirm appropriate model fit. Post hoc comparisons between each plant extract and the solvent control were performed using the posthoc_vsRef function in grafify (version 4.0.1, Shenoy, 2021), with false discovery rate (FDR) correction applied to account for multiple comparisons. We used the ggplot2 package (version 3.4.3, Wickham, 2016) to generate a boxplot with the plant compound as the independent variable and the CI as the dependent variable ( Figure 1C ).

*Classroom Pedagogy*

The experiments in this study were performed over three laboratory sessions by a group of four undergraduates as part of a Course Undergraduate Research Experience independent project. One laboratory session was used to make plant extracts, while the other two sessions were used for chemotaxis assays. These sessions were preceded by training sessions and class research activities where students learned how to extract plant small molecules and conduct *C. elegans* chemotaxis assays (Wu et al., 2026). Weekly homework included reading relevant literature, analysis and visualization of data collected, and writing individual drafts of a journal-style article, which were combined into this article by the student researchers. The instructors then confirmed data results and analysis and provided detailed writing feedback. Assignments were graded as complete/incomplete. All student co-authors approved of the final manuscript.

## Reagents

**Table:** 

Strain	Genotype	Source
CGC1	wild type reference strain formally known as PD1074	Caenorhabditis Genetics Center (CGC) at the University of Minnesota
